# Balancing Chloride and Glucose in Critical Illness: A 10 Year Study on Diluent Strategies and ICU Outcomes

**DOI:** 10.3390/jcm14238573

**Published:** 2025-12-03

**Authors:** Payam Rahimi, Furkan Tontu, Batoul Khoundabi, Sinan Aşar, Çağatay Nuhoğlu, Tuğba Yücel Yenice, Nuri Burkay Soylu, Emral Canan, Zafer Çukurova

**Affiliations:** 1 Bakırköy Dr. Sadi Konuk Training and Research Hospital, 34147 Istanbul, Turkey; nuhoglucagatay@gmail.com (Ç.N.); tugbayucel09@gmail.com (T.Y.Y.); emralc@gmail.com (E.C.); zcukurova@gmail.com (Z.Ç.); 2 Başakşehir Çam and Sakura City Hospital, 34490 Istanbul, Turkey; furkantontu@gmail.com; 3 Iran Helal Institute of Applied-Science and Technology, Tehran 1599665111, Iran; baharkhoundabi@gmail.com; 4 Mardin Training and Research Hospital, 47100 Mardin, Turkey; sinanasaras@gmail.com; 5 Çanakkale State Hospital, 17100 Çanakkale, Turkey; drburkaysoylu@gmail.com

**Keywords:** hyperchloremia, hypochloremia, fluid therapy, mortality

## Abstract

**Background**: High-chloride solutions such as 0.9% saline are widely used for medication dilution in intensive care units (ICUs) and are an underrecognized source of hyperchloremia and acid–base disturbances. Excess chloride reduces the strong ion difference (SID), contributing to hyperchloremic metabolic acidosis and worse clinical outcomes. This study evaluated the impact of replacing isotonic saline with 5% dextrose as a diluent on ICU outcomes in mechanically ventilated patients. **Methods**: In this retrospective cohort study, 4347 adult ICU patients requiring ≥12 h of mechanical ventilation were analyzed across two periods with different diluent strategies (2015–2018: saline-based; 2019–2025: chloride-sparing, dextrose-based). Demographics, comorbidities, illness severity (APACHE II, SOFA), fluid exposure, SID, and laboratory values over the first 48 h were compared. Predictors of mortality were identified using multivariate logistic regression. **Results**: Mortality decreased from 44.6% to 39.2% after adoption of chloride-sparing diluents (absolute reduction 5.4%, *p* = 0.003), despite similar renal function across periods. The later cohort demonstrated significantly higher SID (median 39 vs. 38 mmol/L; *p* < 0.001), lower chloride levels, and more favorable acid–base profiles. In 2015–2018, chloride showed a strong association with mortality (~12–13% increased odds per mmol/L), while in 2019–2025 the association persisted but was attenuated (~2% per mmol/L). SID emerged as a significant marker of improved acid–base balance after the diluent transition. pH remained the most powerful predictor of mortality in both periods. Mean glucose levels increased by ~30–40 mg/dL after switching to dextrose diluents, although insulin requirements did not change. **Conclusions**: Transitioning from chloride-rich to chloride-sparing diluents was associated with reduced ICU mortality and improved acid–base balance, without changes in renal function. However, modestly increased glucose levels highlight the need for strict glycemic monitoring. These findings support chloride-sparing strategies with robust glycemic monitoring in critical care.

## 1. Introduction

Intravenous fluid therapy is a cornerstone of critical care management in intensive care units (ICUs), significantly influencing patient physiology and clinical outcomes [1]. The chloride (Cl) content of these fluids has become a critical focus due to its impact on patient health. High-chloride solutions, such as 0.9% sodium chloride, contain supraphysiological chloride concentrations (154 mmol/L) compared to plasma levels (98–106 mmol/L). This excessive chloride content diminishes the plasma strong ion difference (SID), resulting in hyperchloremic metabolic acidosis [2]. Such acidosis compromises myocardial contractility and tissue oxygen utilization, while chloride-mediated activation of tubuloglomerular feedback triggers renal vasoconstriction, reduces glomerular filtration rate, and increases the risk of acute kidney injury (AKI) [3]. In addition to these renal and metabolic consequences, hyperchloremia has been associated with hemodynamic instability, organ dysfunction, and elevated mortality risk in critically ill patients [4,5]. The SID, defined as the difference between fully dissociated cations and anions, serves as a critical determinant of acid–base balance. A reduction in SID, induced by chloride-rich fluids, through the dissociation of water, leading to hyperchloremic metabolic acidosis [2,6]. Consequently, the SID concept provides a physiological rationale for minimizing chloride exposure in critically ill patients. Excessive chloride contributes to systemic complications. Beyond their application in volume resuscitation, high-chloride solutions, such as isotonic saline, are commonly utilized for medication dilution in the ICU, thereby augmenting chloride exposure. This overlooked source of chloride load may substantially contribute to metabolic disturbances in critically ill patients [7]. In conjunction with the adoption of balanced crystalloids, certain ICUs have sought to minimize chloride exposure by substituting isotonic saline with non-chloride fluids, notably 5% dextrose, for medication dilution [1]. Although this approach may reduce chloride burden and enhance acid–base homeostasis, it raises a significant clinical concern: the potential for hyperglycemia. Importantly, most commonly used ICU medications are compatible with 5% dextrose. Nevertheless, evidence directly associating this practice with patient-centered outcomes, such as mortality or glycemic control, remains limited.

The shift from high-chloride to low-chloride diluent fluids was catalyzed by pivotal studies in the mid-2010s, notably the 2015 SPLIT trial and the 2018 SMART trial, which demonstrated that balanced crystalloids reduced AKI and composite outcomes of death, renal replacement therapy, or persistent renal dysfunction compared to 0.9% saline [8,9]. These findings prompted a paradigm shift, with clinical protocols in many ICUs prioritizing balanced crystalloids over high-chloride fluids by 2015, supported by guidelines like those from the Surviving Sepsis Campaign [10]. This transition reflects a broader recognition of the need to minimize iatrogenic harm through fluid composition that aligns with human physiology [11]. The adoption of low-chloride solutions has been associated with improved acid-base balance, reduced organ dysfunction, and potentially better survival rates, particularly in high-risk populations like septic patients [10,12,13].

This longitudinal study, conducted from 2015 to 2025, examines the clinical effects of substituting isotonic saline with 5% dextrose for medication dilution in mechanically ventilated patients in the ICU. Specifically, the study sought to determine whether this transition was associated with alterations in ICU mortality rates and blood glucose concentrations. By addressing this research gap, our findings offer novel insights into the interplay between chloride restriction and glycemic control, providing practical guidance for optimizing fluid management strategies in critical care practice.

## 2. Materials and Methods

### 2.1. Study Design and Data Collection

After approval by the University of Health Sciences, Bakırköy Dr. Sadi Konuk Training and Research Hospital Ethics Committee with protocol number 2025/200 and decision number 2025-12-06, the study was conducted at Bakırköy Dr. Sadi Konuk Research and Training Hospital ICU. The study included adult patients who were admitted to the ICU for a minimum of 24 h and required mechanical ventilation (MV) for at least 12 h during their ICU stay between January 2015 and July 2025. A total of 4347 patients were analyzed. Pediatric patients, individuals undergoing continuous renal replacement therapy (CRRT) or intermittent hemodialysis, patients diagnosed with COVID-19, non-intubated patients, and those with incomplete or missing key clinical variables were excluded from the study. Patients with an ICU stay of less than 24 h or a mechanical ventilation duration of less than 12 h were excluded from the study. This exclusion was based on the rationale that the metabolic consequences of chloride exposure, such as hyperchloremia and associated acid–base disturbances, typically require sustained fluid administration and are unlikely to manifest in patients with very early ICU mortality or discharge. By excluding these patients, we aimed to focus specifically on the subgroup in which chloride-sparing strategies could have a physiologically significant impact.

Patients were divided into two periods based on changes in diluent fluid management protocols: 2015–2018, when isotonic saline was predominantly used, and 2019–2025, when efforts were made to reduce chloride load by transitioning 5% dextrose solution.

Demographic characteristics (age and sex), clinical data (comorbidities, primary diagnosis, and length of ICU stay, ventilation duration), ICU severity scores (acute physiology and chronic health evaluation II (APACHE II) and sequential organ failure assessment (SOFA) score at admission and the last day of ICU admission, total diluent volume used for medication preparation and the total intravenous fluid volume administered for each patient, laboratory parameters (arterial potential of hydrogen (pH), base excess (BE), bicarbonate (HCO_3_), lactate, Cl, sodium (Na), potassium (K), glucose (Glu), urea, and creatinine, medication data (total doses of insulin) was collected. SID was calculated as a marker of acid–base status according to the Stewart approach. SID was defined as the difference between fully dissociated cations and anions using the equation [2]:

SID = (Na^+^ + K^+^ + Ca^2+^ + Mg^2+^) − (Cl^−^ + lactate)

Data were extracted by utilizing structured query language (SQL) queries from the ImdSoft-Metavision/QlinICU Clinical Decision Support system. The system automatically records patient data from bedside devices on a minute-by-minute basis. These parameters are continuously updated and stored in the database without requiring manual calculation. Minute-level data were extracted and subsequently aggregated into hourly and daily values for analysis. All laboratory parameters were calculated as the mean of measurements taken over the first 48 h of ICU admission.

### 2.2. Diluent Fluid Management Protocols

#### 2.2.1. 2015–2018 Period

During the 2015–2018 period, the standard diluent fluid management protocol in the ICU relied heavily on hyperchloremic fluids, primarily 0.9% isotonic saline (containing 154 mmol/L chloride). The protocol followed conventional ICU practices at the time, with no specific restrictions on chloride load.

#### 2.2.2. 2019–2025 Period

Starting in 2019, our ICU implemented a revised diluent fluid management protocol to minimize chloride load and mitigate the risks associated with hyperchloremia. Non-chloride fluids, such as 5% dextrose, were prioritized for medication dilution to lower overall chloride exposure.

The objective of the study was to evaluate the replacement of isotonic saline with 5% dextrose as a diluent on ICU outcomes in mechanically ventilated patients.

### 2.3. Statistical Analysis

All data were analyzed using SPSS version 27 (IBM Corp., Armonk, NY, USA). Continuous variables were presented as mean ± standard deviation (SD), median and interquartile ranges (IQR) and compared between groups using the independent-samples t-test and Mann–Whitney U test. Categorical variables were expressed as frequencies and percentages and analyzed using the Chi-square and Fisher’s exact test. To further explore predictors of mortality, we performed univariate logistic regression analyses for key physiological and biochemical variables (pH, base excess, HCO_3_, lactate, chloride, potassium, and others), separately for each period. Odds ratios (ORs) with 95% confidence intervals (CIs) were calculated. To facilitate interpretability, ORs were scaled as per 0.1 unit decrease in pH and per 30 mg/dL increase in glucose. Variables with *p* < 0.01 in univariate analysis were entered into multivariate logistic regression models to adjust for potential confounding and to identify independent predictors of mortality. A two-tailed *p*-value < 0.05 was considered statistically significant in all analyses. Chloride changes in three sections were calculated and compared by two durations.

## 3. Results

Among the total participants in the study, there were 4347 patients distributed across two time periods. Specifically, 1038 patients (23.9%) were recorded from 2015 to 2018, and 3309 patients (76.1%) were recorded from 2019 to 2025. Baseline characteristics and comorbidities were comparable between the two cohorts (Table 1). The mean age did not differ (59.3 ± 19.8 vs. 60.9 ± 18.7 years, *p* = 0.11), and the proportion of female patients was identical (37% in 2015–2018 group and 38% in 2019–2025 group). The distribution of comorbidities, including COPD, diabetes, and cardiac disease and illness severity scores including APACHE II and SOFA scores showed no significant differences between groups. Patients in 2019–2025 had shorter ICU stays (16.1 ± 19.5 vs. 10.3 ± 8.9 days, *p* < 0.001) and fewer mechanical ventilation duration (11.2 ± 13.6 vs. 7.8 ± 7.0 days, *p* < 0.001). SID was significantly higher in the 2019–2025 cohort compared to 2015–2018 (39 vs. 38 mmol/L, *p* < 0.001). Fluid management revealed that diluent volume was consistently higher in non-survivors than survivors in both periods, whereas total intravenous fluid volume did not differ significantly. Diluent volume accounted for a greater proportion of total fluid in non-survivors (~39–41%) than in survivors (~29–30%). For total insulin dose, the mean was 0.51 IU/kg/48 h (0.41–0.62) in 2015–2018 and 0.58 IU/kg/48 h (0.44–0.68) in 2019–2025, with no statistically significant difference (*p* = 0.42). Urea levels were 60.6 (38.5–100.0) in 2015–2018 and 58.5 (38.1–95.0) in 2019–2025 (*p* = 0.1). Creatinine levels were 1.1 (0.8–2.1) in 2015–2018 and 1.1 (0.7–1.9) in 2019–2025, which also shows no significant difference (*p* = 0.93). Laboratory values at 48 h (or mean values for patients with a hospital stay shorter than 48 h) demonstrated that survivors in the 2015–2018 cohort exhibited more favorable acid–base and metabolic profiles. Specifically, they had lower chloride, lactate, and glucose levels, as well as higher pH, base excess, and bicarbonate concentrations (all *p* < 0.01). Sodium, potassium, and glucose levels did not differ significantly between survivors and non-survivors. In 2019–2025, differences between survivors and non-survivors were smaller but pH, sodium, and lactate remained significantly different between groups (Table 1).

Per-period analyses of chloride trends (Table S1) showed significant differences between survivors and non-survivors in 2015–2018 (*p* < 0.001), whereas in 2019–2025 the chloride differences were narrowed substantially in 2019–2025.

Variables in Table 1 were included in the multivariate logistic regression analysis. In the 2015–2018 cohort, each 1 mmol/L increase in serum chloride was associated with a 13% increase in the odds of mortality. In the 2019–2025 cohort, each 1 mmol/L increase linked to only a 2% increase in mortality odds. In both periods, higher pH, were significantly associated with survival: each 0.1-unit increase in pH was associated with a ~53% decrease in mortality risk in 2015–2018 and ~79% in 2019–2025. Lactate and potassium also emerged as consistent and robust predictors of poor outcome in 2015–2018 group. Each 1 mmol/L increase in lactate was associated with a ~32% rise in mortality risk, while each 1 mmol/L increase in potassium increased mortality odds by 23%. In the 2015–2018 cohort, base excess and bicarbonate emerged as significant protective factors, with higher values associated with reduced mortality risk. Glucose exhibited a modest but statistically significant association with increased mortality. In contrast, in the 2019–2025 cohort none of the laboratory parameters (potassium, lactate, base excess, bicarbonate, or glucose) retained statistical significance in the multivariable analysis (Table 2).

Table 3 summarizes multivariate logistic regression models for mortality in the two periods. In 2015–2018, both chloride and pH remained significant in multivariate models: higher chloride increased mortality risk (≈12% per mmol/L), while higher pH was strongly protective (~50% per 0.1 unit increase). In the Cl + K model, both variables were also significant, with Cl increasing odds by 13% and K by 26%. In the 2019–2025 cohort the combined lactate + potassium model demonstrated that lactate remained an independent predictor of mortality (*p* = 0.004) while potassium did not (*p* = 0.465). In the 2019–2025 cohort, when chloride was modeled together with lactate, both variables were small but independently associated with mortality (chloride increasing and lactate decreasing the odds of death), whereas in the chloride–glucose model only chloride remained a small but significant predictor.

A fully adjusted logistic regression model including demographic, clinical, and biochemical variables is presented in Supplementary Table S2. In this model, female sex (OR 1.35, *p* = 0.006), higher APACHE II and SOFA scores, lower pH, higher lactate, higher chloride and glucose levels were independently associated with increased mortality. In contrast, primary diagnosis and comorbidities were not significant predictors. Patients admitted in 2015–2018 had more than double the odds of death compared with those admitted in 2019–2025 (OR 1.57, *p* = 0.001). Potassium did not retain statistical significance after full adjustment.

## 4. Discussion

This decade-long retrospective cohort study of 4347 mechanically ventilated ICU patients demonstrates a significant association between the transition from high-chloride (isotonic saline) to low-chloride (5% dextrose) medication diluents and improved clinical outcomes. This shift, prompted by evidence from trials like SPLIT and SMART [8,9], reduced mean chloride levels by 3–4 mmol/L in the first 48 h, highlighting medication diluents as a substantial, often overlooked contributor to chloride burden in critical care [7]. Our focus on mechanically ventilated patients, who are at heightened risk for fluid-induced metabolic derangements, addresses a key gap in prior research. The observed disparity in total diluent volume, which was consistently higher in non-survivors compared to survivors, merits careful consideration. This finding likely reflects the increased medication requirements and greater treatment intensity in patients with more severe illness. Non-survivors received a larger proportion of their total intravenous fluid as diluent, indicating that the cumulative medication burden and underlying disease severity contributed to this difference. Consequently, while chloride content remains a critical modifiable factor, the total diluent volume also appears to serve as a surrogate marker of disease severity and therapeutic complexity.

Our findings are consistent with growing evidence that medication diluents are a major, often underrecognized, source of chloride exposure in the ICU. Aoyagi et al. demonstrated that using dextrose instead of saline as a diluent reduced hypernatremia and hyperchloremia, without increasing hyperglycemia, AKI, or mortality [14]. Magee et al. provided prospective evidence that diluents accounted for 63% of IV fluid volume, and that switching to 5% dextrose in water halved the incidence of hyperchloremia (from 17.9% to 10.5%, adjusted OR 0.50), again without worsening glycemia or renal outcomes [15]. These findings align mechanistically with chloride role in reducing the SID, leading to hyperchloremic acidosis and potential renal vasoconstriction [4,5]. In our cohort, the lack of significant changes in urea/creatinine levels suggests that mortality benefits may stem more from acid-base stabilization than renal protection, possibly due to our population’s baseline comorbidities or concurrent therapies. Finally, a 2022 meta-analysis by Alsohimi et al., including >1500 patients, confirmed that replacing saline with 5% dextrose in water significantly reduced the risk of hypernatremia and hyperchloremia without increasing AKI, hyperglycemia, or mortality, and may shorten ICU length of stay [16]. Together, these data align with our results and underscore that protocol-level substitution of saline with dextrose is safe and effective in reducing dyschloremia.

The significance of the SID in our analysis underscores its value as an integrative marker of acid–base physiology. The higher SID observed in the 2019–2025 cohort reflects the substantial reduction in chloride exposure achieved through the use of 5% dextrose as the medication diluent. This improvement in SID provides mechanistic support for the observed enhancement in acid–base homeostasis and its association with better clinical outcomes. Prior studies have established that a low strong ion difference (SID), driven predominantly by hyperchloremia, is associated with metabolic acidosis and increased mortality in critically ill patients [17,18]. Our findings extend this evidence by demonstrating that the observed increase in SID following the transition to chloride-sparing diluents was accompanied by improved acid–base stability and reduced mortality. Incorporating SID into the evaluation of fluid management strategies provides a more physiologically comprehensive assessment of iatrogenic acid–base disturbances than reliance on serum chloride concentration alone.

A notable trade-off was a ~30–40 mg/dL rise in mean blood glucose after the dextrose transition, despite unchanged total insulin doses (*p* = 0.42). This may reflect unadjusted protocols for dextrose-induced glucose loads or patient factors like insulin resistance in sicker cohorts. While prior studies reported no hyperglycemia worsening [14,15,16], our results highlight the need for enhanced monitoring and dynamic insulin titration to prevent attenuation of survival gains. Future protocols could integrate continuous glucose monitoring to balance chloride restriction with glycemic control.

pH, lactate, potassium, and chloride were the strongest predictors of mortality, with pH emerging as the strongest predictor. This reflects the integration of multiple derangements, including lactate-driven acidosis and chloride-induced SID reductions [19,20]. Chloride remained a small but significant predictor of mortality in 2019–2025 (OR ≈ 1.02 per mmol/L), although the effect size was markedly reduced compared to 2015–2018 (OR ≈ 1.12–1.13). The large sample enabled robust regression models, improving on the previous manuscript. Brown et al. emphasize pH and lactate as key markers [19]. Furthermore, survivors exhibited higher pH, base excess (BE), and bicarbonate (HCO_3_) levels 2015–2018 group compared to non-survivors (all *p* < 0.001), with greater improvements in 2019–2025. This reflects improved acid-base homeostasis with low-chloride fluids, particularly in mechanically ventilated patients at risk of hyperchloremic metabolic acidosis [20]. Analyses suggested lower chloride levels in survivors in 2015–2018 group and supplemental per-period evaluation (Table S1) demonstrated that differences were statistically significant once 48 h changes were considered. In contrast, our fully adjusted logistic regression model (Table S2) confirmed chloride as an independent predictor of mortality, consistent with its mechanistic role in acid–base homeostasis. The study by Hammond et al. supports this, reporting reduced hyperchloremic acidosis with balanced crystalloids (RR 0.78, 95% CI 0.66–0.91) [13]. The previous manuscript noted similar trends but lacked the current study’s detailed chloride monitoring and large sample. The interplay of chloride and HCO_3_, as noted by Tan et al. in heart failure patients [21], underscores our findings of pH and HCO_3_ as strong survival predictors.

The strengths of this study significantly enhance its contribution to the critical care literature. The inclusion of a large cohort of 4347 patients over 10 years (2015–2025) provides substantial statistical power, enabling robust analyses of mortality, acid-base balance, and other outcomes, and markedly improving the generalizability of findings compared to the smaller sample size in the previous studies in the literature. Additionally, the study’s dedicated focus on mechanically ventilated patients addresses a critical gap in the literature, as this high-risk group is particularly susceptible to fluid-related complications such as hyperchloremic metabolic acidosis and organ dysfunction, thereby strengthening the relevance of the findings for ICU populations with prolonged ventilation needs. Furthermore, the comprehensive chloride monitoring implemented in the 2019–2025 period, which involved frequent serum chloride assessments and tailored fluid therapy, represents a significant advancement over the previous manuscript’s less consistent monitoring approach, providing a clearer link between chloride levels and clinical outcomes. Nonetheless, the large sample and detailed 48 h laboratory averaging provide granular insights absent in smaller studies.

Several limitations should be acknowledged. First, its retrospective, single-center design may limit generalizability and is susceptible to unmeasured confounders such as evolving sepsis guidelines [10], ventilatory strategies, or case-mix changes during the 10-year period. Second, although we consistently applied diluent protocols, drug-level adherence and certain granular clinical practices could not be fully captured, which may have influenced outcomes.

Our findings indicate an association, rather than definitive causality, between diluent choice and survival outcomes. Although detailed drug-level adherence could not be captured, in practice, all medications were diluted with isotonic saline during the 2015–2018 period, and after 2019, nearly all were diluted with 5% dextrose, with the exception of 16 patients receiving phenytoin, which required isotonic saline dilution due to compatibility guidelines. This high level of consistency strongly supports adherence to the protocol. However, we acknowledge that the retrospective study design precludes definitive establishment of causality. Although chloride-sparing strategies may have contributed to improved acid–base profiles, patient outcomes are concurrently influenced by multiple ICU practices and underlying illness severity. Propensity score matching or adjustment for these factors in future analyses could strengthen causal inferences.

Our findings advocate for widespread adoption of chloride-sparing diluents like 5% dextrose in ICUs, potentially reducing mortality by 12–13% in ventilated patients when paired with optimized glycemic control. Multicenter randomized trials are needed to confirm causality, explore subgroup effects (e.g., in septic vs. surgical patients), and assess long-term outcomes.

## 5. Conclusions

Replacing isotonic saline with 5% dextrose for drug dilution in mechanically ventilated ICU patients was associated with reduced mortality. No clinically meaningful differences were observed in renal function markers, such as serum urea and creatinine, suggesting that the primary benefit of chloride restriction pertained to enhanced acid–base stability rather than renal protection. However, this approach was accompanied by increased blood glucose concentrations, which modestly elevated mortality risk. These findings support routine use of chloride-sparing diluents, such as 5% dextrose, provided they are paired with structured glycemic monitoring and insulin adjustment protocols. Overall, our results suggest that incorporating chloride-sparing strategies in the ICU may enhance survival outcomes while preserving patient safety, provided they are integrated with optimized glucose management.

## Figures and Tables

**Table 1:** Baseline demographic, clinical, and 48 h biochemical characteristics of ICU patients across two periods (2015–2018 vs. 2019–2025).

		2015–2018 *n*: 1038	*p* Value	2019–2025 *n*: 3309	*p* Value
Primary Diagnosis	Abdominal	194		634	0.736
	Cardiac	97		323	0.890
	Respiratory	244		728	0.310
	Metabolic	45		147	0.952
	Neurologic	281		907	0.831
	Renal	139		449	0.884
	Other	38		121	0.930
Comorbidities	CAD	30		90	0.854
	COPD	278		892	0.912
	CRF	7		32	0.494
	CVD	43		91	0.082
	DM	57		160	0.937
	HF	50		155	0.860
	HT	101		326	0.908
	LF	22		64	0.805
Demographic	Age (year)	59.3 ± 19.8		60.9 ± 18.7	0.115
	Female *n* (%)	387 (37.3%)		1264 (38.2%)	0.596
	LOS in ICU (day)	16.1 ± 19.5		10.3 ± 8.9	<0.001 *
	APACHE II	24.4 ± 8.3		23.8 ± 7.9	0.237
	SOFA Initial	8.6 ± 3.8		8.9 ± 3.6	0.360
	SOFA Final	7.5 ± 4.7		8.0 ± 4.9	0.598
	MV Duration (day)	11.2 ± 13.6		7.8 ± 7.0	<0.001 *
	SID (mmol/L)	38 (33.8–42.8)		39 (36–43.1)	<0.001 *
Total Diluent Volume (L)	Survived	1.81 (0.21–4.43)	<0.001 *	1.85 (0.2–4.4)	<0.001 *
	Non-Survived	2.73 (0.83–6.21)		2.54 (0.78–6.18)	
Total Intravenous Fluid Volume (L)	Survived	6.2 (0.8–10.7)	0.79	6.2 (0.6–10.4)	0.54
	Non-Survived	6.6 (0.5–12.0)		6.5 (0.4–11.4)	
Laboratory values					
Cl (mmol/L)	Survived	101.6 ± 5.1	<0.001 *	100.6 ± 6.2	0.303
	Non-Survived	107.9 ± 8.9		100.8 ± 6.3	
Na (mmol/L)	Survived	140.7 ± 5.7	0.783	138.9 ± 6.1	0.004 *
	Non-Survived	141.3 ± 8.1		139.7 ± 6.2	
K (mmol/L)	Survived	4.1 ± 0.6	0.010 *	4.3 ± 0.6	0.625
	Non-Survived	4.2 ± 0.7		4.3 ± 0.6	
pH	Survived	7.4 ± 0.1	<0.001 *	7.4 ± 0.1	0.004 *
	Non-Survived	7.3 ± 0.1		7.3 ± 0.1	
BE (mmol/L)	Survived	0.2 ± 4.9	<0.001 *	−1.1 ± 5.9	0.266
	Non-Survived	−2.2 ± 5.5		−1.1 ± 5.2	
HCO_3_ (mmol/L	Survived	19.7 ± 5.6	0.001 *	23.6 ± 4.8	0.996
	Non-Survived	18.4 ± 5.1		23.3 ± 4.3	
Lactate (mmol/L)	Survived	2.0 ± 1.2	<0.001 *	2.6 ± 2.7	0.008 *
	Non-Survived	2.6 ± 1.8		2.9 ± 2.4	
Glucose (mg/dL)	Survived	150.2 ± 45.9	0.019 *	179.4 ± 58.6	0.955
	Non-Survived	158.2 ± 53.4		177.9 ± 53.9	

*: statistically significant at 0.05 level. All parameters are presented as mean ± SD, except total intravenous fluid volume and diluent volume, which are expressed as median (IQR). SD: Standard deviation, CAD: Coronary artery disease, COPD: Chronic obstructive pulmonary disease, CRF: Chronic renal failure defined as end-stage renal disease on maintenance hemodialysis prior to ICU admission, CVD: Cerebrovascular disease, DM: Diabetes mellitus, HF: Heart failure, HT: Hypertension, LF: Liver failure, LOS in ICU: Length of stay in intensive care unit, APACHE: Acute physiology and chronic health evaluation, SOFA: Sequential organ failure assessment, SID: Strong ion difference, MV: Mechanical ventilation, Cl: Chloride, Na: Sodium, K: Potassium, pH: Potential of hydrogen, BE: Base excess, HCO_3_: Bicarbonate.

**Table 2:** Univariate Logistic Regression for predictors of ICU Mortality (2015–2018 and 2019–2025).

Variable	2015–2018 B	Exp(B) (Change in Odds of Death)	*p* Value	2019–2025 B	Exp(B) (Change in Odds of Death)	*p* Value
Cl	0.124	1.132 (↑13%)	<0.001 *	0.023	1.023 (↑2%)	<0.001 *
K	0.211	1.234 (↑23%)	0.031 *	0.023	1.024 (↑2%)	0.680
pH	−7.571	0.469 (↓53%) ^$^	<0.001 *	−1.548	0.213 (↓79%)	<0.001 *
BE	−0.085	0.918 (↓8%)	<0.001 *	0.001	1.000 (0%)	0.973
HCO_3_	−0.044	0.957 (↓4%)	<0.001 *	−0.012	0.988 (↓1%)	0.117
Lactate	0.279	1.322 (↑32%)	<0.001 *	−0.043	0.958 (↓4%)	0.005
Glucose	0.003	1.094 (↑1%) ^$^	0.010 *	0.001	1.000 (0%)	0.447

*: significant at 0.05 level, ^$^: OR was calculated for 0.1 mg/dL decrease in pH and 30 mg/dL increase in glucose. Cl: Chloride, K: Potassium, pH: Potential of hydrogen, BE: Base excess, HCO_3_: Bicarbonate.

**Table 3:** Multivariate Logistic Regression Models of Biochemical predictors of ICU mortality (2015–2018 and 2019–2025).

Model Variables	Period	B Coefficient(s)	Exp(B) (Change in Odds)	*p*-Value(s)
Cl + pH	2015–2018	Cl: 0.113, pH: −6.849	Cl: 1.120 (↑12%), pH: 0.504 (↓50%) ^$^	<0.001 *
Cl + K	2015–2018	Cl: 0.124, K: 0.231	Cl: 1.132 (↑13%), K: 1.260 (↑26%)	<0.001 */0.033
Lactate + K	2019–2025	Lactate: −0.044, K: 0.042	Lactate: 0.957 (↓4%), K: 1.043 (↑4%)	0.004 */0.465
Cl + Lactate	2019–2025	Cl: 0.022, Lactate: −0.039	Cl: 1.022 (↑2%), Lactate: 0.962 (↓4%)	<0.001 */0.011 *
Cl + Glu	2019–2025	Cl: 0.023, Glu: −0.001	Cl: 1.023 (↑2%), Glu: 0.970 (↓3%) ^$^	<0.001 */0.367

*: significant at 0.05 level, ^$^: OR was calculated for 0.1 mg/dL decrease in pH and 30 mg/dL increase in glucose. Cl: Chloride, pH: Potential of hydrogen, K: Potassium, Glu: Glucose.

## Data Availability

De-identified patient data and analysis code supporting the findings of this study are available from the corresponding author upon request. Access will require approval by the ICU chief of Bakırköy Dr. Sadi Konuk Training and Research Hospital, in accordance with local regulations on patient data protection.

## Supplementary Materials

The following supporting information can be downloaded at: https://www.mdpi.com/article/10.3390/jcm14238573/s1. Table S1: Per-period changes in serum chloride (mmol/L) over the first and last 48 h of ICU admission in survivors and non-survivors; Table S2: Multivariate logistic regression of demographic, clinical, and biochemical predictors of ICU mortality (2015–2025).

## Abbreviations

AKI	Acute Kidney Injury
APACHE II	Acute Physiology and Chronic Health Evaluation II
BE	Base Excess
Cl	Chloride
CRRT	Continuous Renal Replacement Therapy
D5W	5% Dextrose in Water
Glu	Glucose
HCO_3_^−^	Bicarbonate
ICU	Intensive Care Unit
IQR	Interquartile Range
IV	Intravenous
K^+^	Potassium
MV	Mechanical Ventilation
Na^+^	Sodium
OR	Odds Ratio
pH	Potential of Hydrogen
RR	Relative Risk
SD	Standard Deviation
SE	Standard Error
SID	Strong Ion Difference
SOFA	Sequential Organ Failure Assessment

## References

[1] Silva C., Marcos P. (2025). Intravenous fluid therapy: Essential components and key considerations. Porto Biomed. J..

[2] Kellum J.A., Elbers P.W. (2009). Stewart’s Textbook of Acid-Base.

[3] Tontu F. (2025). Fluid Selection in Renal Transplant Patients: Considerations for Hyperkalemia Management. Turk. J. Anaesthesiol. Reanim..

[4] Duan Y., Yin Y., Yue J., Zhu W., Yang X., Ma Y., Wan X., Yang Y. (2025). Is restriction of intravenous fluid beneficial for septic shock in ICU patients? A meta-analysis of randomized controlled trials. BMC Anesthesiol..

[5] Sudheer N., James J.V., Sudheer Jr N., James Sr J. (2024). Fluid Management in Critical Care: New Insights Into Optimal Fluid Therapy. Cureus.

[6] Ciabattoni A., Chiumello D., Mancusi S., Pozzi T., Monte A., Rocco C., Coppola S. (2025). Acid-Base Status in Critically Ill Patients: Physicochemical vs. Traditional Approach. J. Clin. Med..

[7] Choo W.P., Groeneveld A.B., Driessen R.H., Swart E.L. (2014). Normal saline to dilute parenteral drugs and to keep catheters open is a major and preventable source of hypernatremia acquired in the intensive care unit. J. Crit. Care.

[8] Young P., Bailey M., Beasley R., Henderson S., Mackle D., McArthur C., McGuinness S., Mehrtens J., Myburgh J., Psirides A. (2015). Effect of a buffered crystalloid solution vs saline on acute kidney injury among patients in the intensive care unit: The SPLIT randomized clinical trial. JAMA.

[9] Brown R.M., Wang L., Coston T.D., Krishnan N.I., Casey J.D., Wanderer J.P., Ehrenfeld J.M., Byrne D.W., Stollings J.L., Siew E.D. (2019). Balanced crystalloids versus saline in sepsis. A secondary analysis of the SMART clinical trial. Am. J. Respir. Crit. Care Med..

[10] Rhodes A., Evans L.E., Alhazzani W., Levy M.M., Antonelli M., Ferrer R., Kumar A., Sevransky J.E., Sprung C.L., Nunnally M.E. (2017). Surviving sepsis campaign: International guidelines for management of sepsis and septic shock: 2016. Intensive Care Med..

[11] Arabi Y.M., Belley-Cote E., Carsetti A., De Backer D., Donadello K., Juffermans N.P., Hammond N., Laake J.H., Liu D., Maitland K. (2024). European Society of Intensive Care Medicine clinical practice guideline on fluid therapy in adult critically ill patients. Part 1: The choice of resuscitation fluids. Intensive Care Med..

[12] Semler M.W., Self W.H., Wanderer J.P., Ehrenfeld J.M., Wang L., Byrne D.W., Stollings J.L., Kumar A.B., Hughes C.G., Hernandez A. (2018). Balanced crystalloids versus saline in critically ill adults. N. Engl. J. Med..

[13] Hammond N.E., Zampieri F.G., Di Tanna G.L., Garside T., Adigbli D., Cavalcanti A.B., Machado F.R., Micallef S., Myburgh J., Ramanan M. (2022). Balanced crystalloids versus saline in critically ill adults—A systematic review with meta-analysis. NEJM Evid..

[14] Aoyagi Y., Yoshida T., Uchino S., Takinami M., Uezono S. (2020). Saline versus 5% dextrose in water as a drug diluent for critically ill patients: A retrospective cohort study. J. Intensive Care.

[15] Magee C.A., Bastin M.L.T.P., Laine M.E.P., Bissell B.D.P., Howington G.T., Moran P.R., McCleary E.J.P., Owen G.D., Kane L.E., Higdon E.A.P. (2018). Insidious Harm of Medication Diluents as a Contributor to Cumulative Volume and Hyperchloremia: A Prospective, Open-Label, Sequential Period Pilot Study. Crit. Care Med..

[16] Alsohimi S., Almagthali A.G., Eljaaly K., Korayem G.B., Al Sulaiman K., Aljuhani O. (2022). 0.9% Sodium Chloride Versus Dextrose 5% in Water Safety as Medication’s Diluents in Critically Ill Patients: Meta-Analysis of Observational Studies. Saudi Crit. Care J..

[17] Altun H.I., Altun G., Altas O.F., Aran G. (2023). Prognostic Significance of the Strong Ion Gap in Patients in Medical and Surgical Intensive Care Units. Cureus.

[18] Gil H.W., Hong M., Lee H., Cho N.J., Lee E.Y., Park S. (2021). Impact of Acid-Base Status on Mortality in Patients with Acute Pesticide Poisoning. Toxics.

[19] Brown R.M., Wang L., Casey J.D., Jackson K.E., Self W.H., Rice T.W., Semler M.W. (2020). Reply to Gueret et al. and to Hammond et al. Am. J. Respir. Crit. Care Med..

[20] Sagar N., Lohiya S., Sagar A.N. (2024). A comprehensive review of chloride management in critically ill patients. Cureus.

[21] Tan Z., Liu Y., Hong K. (2024). The association between serum chloride and mortality in ICU patients with heart failure: The impact of bicarbonate. Int. J. Cardiol..

