# Supplementary material for: Balancing Chloride and Glucose in Critical Illness: A 10 Year Study on Diluent Strategies and ICU Outcomes
Source: J Clin Med. 2025 Dec 3;14(23):8573. doi: 10.3390/jcm14238573 (PMC12693138; doi:10.3390/jcm14238573)
Supplement: Supplementary file 1 [file jcm-14-08573-s001.zip › jcm-3852735-supplementary.pdf]

Table S1. Per-period changes in serum chloride (mmol/L) over the first and last 48 hours of ICU admission in survivors and non-survivors.

Duration	Cl changes	survival status	Mean	SD	SE	p value
2015-2018	Averaged 48_first	Survived	0.730	7.104	0.308	<0.001*
		Non-Survived	6.995	11.102	0.492	
	Last_ Averaged 48	Survived	1.465	8.291	0.360	<0.001*
		Non-Survived	-3.487	11.877	0.526	
	Last_ first	Survived	2.195	9.924	0.431	0.045*
		Non-Survived	3.508	11.457	0.507	
2019-2025	Averaged 48_first	Survived	-1.601	7.069	0.154	0.781
		Non-Survived	-1.292	6.498	0.187	
	Last_ Averaged 48	Survived	-0.246	8.426	0.183	0.390
		Non-Survived	-0.775	8.965	0.258	
	Last_ first	Survived	-1.847	9.883	0.215	0.757
		Non-Survived	-2.068	10.196	0.293	

*: Significant at 0.05 level. SD: Standard deviation, SE: Standard, Averaged 48_first: Mean chloride during first 48 h; Last_Averaged 48: Mean chloride during last 48 h; Last_first: Change between last and first 48 h, Cl: Chloride,

Table S2. Multivariate logistic regression of demographic, clinical, and biochemical predictors of ICU mortality (2015–2025).

**Model Variables**	B	SE	Wald	P-value	Exp(B)	95% CI for Exp(B)	
						Lower	Upper
Age	-0.006	0.003	4.107	0.043*	0.994	0.988	1.000
Sex (Female)	0.301	0.109	7.617	0.006*	1.351	1.091	1.673
Disease (Lung)			4.227	0.517			
Disease (Abdominal)	-0.194	0.184	1.123	0.289	0.823	0.575	1.180
Disease (Cardiac)	-0.048	0.235	0.042	0.838	0.953	0.601	1.511
Disease (Other)	-0.658	0.408	2.604	0.107	0.518	0.233	1.152
Disease (Neurologic)	-0.083	0.165	0.255	0.614	0.920	0.666	1.271
Disease (Renal)	-0.323	0.264	1.493	0.222	0.724	0.431	1.215
comorbidity (None)			2.684	0.612			
Comorbidity (Other)	0.175	0.245	0.515	0.473	1.192	0.738	1.924
Comorbidity (Cardiac)	0.295	0.185	2.546	0.111	1.344	0.935	1.931
Comorbidity (Respiratory)	0.136	0.153	0.791	0.374	1.146	0.849	1.547
Comorbidity (Gland)	0.192	0.199	0.930	0.335	1.212	0.820	1.792
APACHE II	0.024	0.008	9.220	0.002*	1.024	1.008	1.040
SOFA	0.447	0.017	732.320	<0.001*	1.564	1.514	1.616
pH	-7.354	0.767	91.872	<0.001*	0.001	0.000	0.003
Lactate	0.110	0.034	10.238	0.001*	1.116	1.043	1.194
Cl	0.155	0.010	257.273	<0.001*	1.168	1.146	1.190
Glu	0.002	0.001	4.297	0.038*	1.002	1.000	1.004
K	-0.084	0.089	0.887	0.346	0.919	0.771	1.095
Time (2015-2018)	0.447	0.134	11.110	0.001*	1.563	1.202	2.033
Constant	1.024	5.849	0.059	0.723	2.771		

*: significant at 0.05 level, OR: Odds ratio, SE: Standard error, CI: Confidence Interval, Reference groups: male sex, lung disease, absence of comorbidity, and admission period 2019–2025, Apache II: Acute physiology and chronic health evaluation, SOFA: Sequential organ failure assessment, Cl: chloride, Glu: glucose, K: potassium.
